# The effect of psychological stress on iron absorption in rats

**DOI:** 10.1186/1471-230X-9-83

**Published:** 2009-11-13

**Authors:** Jianbo Chen, Hui Shen, Chengjie Chen, Wanyin Wang, Siyu Yu, Min Zhao, Min Li

**Affiliations:** 1 Department of Military Hygiene, Second Military Medical University, 800 Xiangyin Road, Shanghai 200433, PR China

## Abstract

**Background:**

Psychological stress (PS) is recognized as an important pathogenic factor which leads to metabolism disorder in many diseases. Previous studies have shown that systemic iron homeostasis in mammalians was changed under specific stress conditions.

**Methods:**

In present study, we used communication box to create psychological stress model and investigated the iron apparent absorption, iron accumulation in the apical poles of villous enterocytes and protein expressions of ferroportin 1 (FPN1), ferritin, divalent metal transporter 1 (DMT1).

**Results:**

Our study showed that iron apparent absorption decreased and iron significantly accumulated in the apical poles of villous enterocytes in 3 d and 7 d PS groups. The expression of intestinal FPN1 in 3 d and 7 d PS groups was lower than that of control, while the change of intestinal ferritin was opposite. However, the expression of DMT1 did not change.

**Conclusion:**

These results demonstrate that PS can decrease iron absorption in rats, which might be related to regulation expression of iron transporters.

## Background

Iron is essential for almost all forms of life but is toxic in excess and therefore body iron levels must be tightly regulated [1-3]. As the body cannot actively excrete iron, the body iron content must be regulated at the point of absorption across the mature villous enterocytes of duodenum [4]. Our knowledge of the mechanisms that regulate iron absorption has recently advanced dramatically. Two iron transporters, divalent metal transporter 1 (DMT1) and ferroportin 1 (FPN1), are critical for intestinal iron absorption. DMT1 is a transmembrane protein that transports ferrous iron across the apical membrane of intestinal epithelial cells [5], whereas FPN1 is an iron exporter located on the basolateral membrane [6].

Our previous study demonstrated that after repeated psychological stress (PS) exposure, the serum iron level decreases and erythropoiesis gets inhibited while iron uptake is normal [7]. However, the molecular mechanisms how PS leads to iron mal-regulation are not well known. In present study, we investigated the effect of PS on iron absorption and the expressions of iron transporters in the small intestine so as to elucidate possible underlying mechanism.

## Methods

### Animals and PS exposure

The animals were fed and treated as described [8,9]. Thirty male Sprague-Dawley (SD) rats (Shanghai-BK Co., Ltd. Shanghai, China), weighing 120 ± 5 g, were housed individually in cages at a temperature of 24 ± 1°C, a humidity of 55 ± 5% in a 12-h light/dark cycle, and were given normal chow and free access to water. The iron content in diet was 35 mg/kg. After 7 d adaption, the rats were divided into two groups randomly: control group and PS group. Each group was subdivided into three subgroups: 1 d group, 3 d group and 7 d group (5 rats in every subgroup). Each animal was used only once in the experiment. All animal treatments were strictly in accordance with international ethical guidelines and the National Institutes of Health Guide concerning the Care and Use of Laboratory Animals, and the experiments were carried out with the approval of the Committee of Experimental Animal Administration of the University.

PS model was created in rats as described previously [8]. Briefly, a communication box was divided into Room A and Room B with a transparent acrylic board. Room A included 10 little rooms with a plastic board-covered floor and Room B included 10 little rooms with a metal grid-exposed floor for electric insulation. Rats in Room B were randomly given electrical shock (0.6 mA for 1 s) for 30 min (60 times) through the floor and exhibited nociceptive stimulation-evoked responses, such as jumping up, defecation and crying; rats in Room A were only exposed to the responses of rats in Room B to establish PS model. PS was given to rats for 30 min every morning (10:00-10:30) for 7 days. At the end of the exposure, the rats were kept in the cages for another 4 min before they were taken out. Animals in the control group were only kept in the cages for 4 min without receiving any stress.

At the end of PS exposure all rats were deeply anesthetized by intraperitoneal (i.p.) injection of 7% chloral hydrate [8,9]. Blood samples were collected from the heart followed by centrifuging at 3 000 g for 15 min, and the supernatants were obtained and stored at -80°C for futher determination. Then the rats were perfused through the left cardiac ventricle with ice-cold phosphate buffered saline (PBS; pH 7.4) to flush out the plasma. Hypothalamus and duodenum were quickly removed and snap frozen in liquid nitrogen, and kept in a -80°C freezer till use. Perfused duodenum were sectioned at 30 μm on a sliding microtome into free-floating tissue sections.

Contents of noradrenalin (NE) in hypothalamus, corticosterone (CORT) and adrenocorticotropic hormone (ACTH) in serum were analyzed using commercially available ELISA kits (R&D Systems, Inc., USA). Coefficient of variation (CV) values for NE, CORT and ACTH were 13%, 15% and 11% separately.

### Iron absorption measurement

The fecal samples were collected, dried and weighted daily. Urine was not collected or analyzed for iron content, because iron excretion in urine was assumed to be negligible. The diets were changed daily. Samples were stored in polypropylene vials and kept at -20°C before analysis.

To insure accurate collection of feces at day 1, day 3 and day 7 during PS, rat was put into a plastic cage independently to prevent any loss of feces. Before food intake, animals were given carmine red by intragastric administration. Feces were collected between two red feces (not including the second). Apparent absorption of iron was expressed as (iron content in food - iron content in feces)/iron content in food × 100%.

Iron contents in the feces were measured using inductively coupled plasma mass spectrometry (ICP-MS). Before analysis, fecal samples were weighted and then desiccated in an oven at 90°C. The dried samples (0.2 g) were dissolved in 10 ml of 18 N HNO_3 _by incubation at 90°C for 24 h and then acid was evaporated at 58°C. Samples were resuspended in 5 ml of 2 N HNO_3 _and analyzed by ICP-MS using 2 N HNO_3 _as the blank.

### Membrane and soluble protein preparation

The frozen duodenum samples were homogenized in 5 ml HEPES-EDTA buffer [20 mmol Hepes/L, pH 7.4; 1 mmol EDTA/L; 250 mmol sucrose/L; a protease inhibitor mixture containing 4-(2-aminoethyl)benzenesulfonyl fluoride, trans-epoxysuccinyl-L-leucylsmido(4-guanidino)butane, bestatin, leupeptin, aprotinin, and sodium EDTA (Sigma)].. The homogenate was centrifuged at 500 g for 5 min at 4°C. The supernatant fluid was then centrifuged at 100 000 g for 30 min at 4°C. The supernatant fluid (soluble protein) was collected for ferritin protein determination, and the crude membrane fraction was resuspended in 0.3 ml homogenization buffer for DMT1 and FPN1 protein determination. Samples were stored at -70°C till analysis. Protein concentrations of the soluble and membrane fractions were quantified by the method of Lowry [10].

### Western blotting analysis of DMT1, FPN1 and ferritin expressions

Aliquots of the lysates containing 50 μg of protein were diluted in 2× sample buffer [50 mM Tris, pH 6.8, 2% sodium dodecyl sulfate (SDS), 10% glycerol, 0.1% bromophenol blue, and 5% μ-mercaptoethanol] and heated for 5 min at 95°C before SDS-polyacrylamide gel electrophoresis (PAGE) on a 10% gel and subsequently transferred to a pure nitrocellulose membrane under conditions 200 mA during 120 min. The blots were blocked with 5% nonfat dry milk in Tris-buffered saline with Tween 20 (TBS-T; 20 mM Tris-Cl, pH 7.6, 137 mM NaCl, 0.1% Tween 20) for 2 h at room temperature. Proteins were incubated overnight at 4°C with a primary antibody against DMT1 (1:1000, ADI), FPN1 (1:1000, ADI), ferritin (1:5000, Sigma), or β-actin (1:10000, Sigma). The immunoreactive bands were detected by goat polyclonal anti-rabbit-HRP antibody (Santa Cruz, CA). The blots were developed by incubation in ECL chemiluminescence reagent (Amersham Life Science, Arlington Heights, IL, USA) and subsequently exposed to BioMax Light Film (Eastman Kodak Co., USA). Processed blots were quantified by using the BandScan 5.0 software.

### Perl's staining

For Perl's staining, sections were processed through a series of graded alcohols, into xylene, and rehydrated back to water. Sections were incubated in a 1:1 solution of 2% HCl and potassium ferrocyanide (Sigma) for 30 min and rinsed in water. Sections were counterstained with Neutral Red, dehydrated in increasing concentrations of ethanol, cleared in xylene, and mounted on slides.

### Statistical analysis

All results were expressed as means ± SD. Statistical analysis was carried out by using SPSS 11.0. One-way ANOVA, correcting for differences in sample variance, was used to determine whether differences were statistically significant in groups. A *P *value less than 0.05 was a considered statistically significant difference.

## Results

### PS induced changes of CORT, ACTH and NE

After 7 d PS, NE in hypothalamus, CORT and ACTH in serum all increased significantly (Table 1)

**Table 1:** CORT and ACTH in serum and NE in hypothalamus in control and PS groups (means ± SD, n = 5)

	CORT(ng/ml)	ACTH(pg/ml)	NE(ng/g)
Control	355.05 ± 35.53	330.25 ± 33.38	0.0527 ± 0.011
PS	426.13 ± 47.64*	393.56 ± 36.51*	0.0896 ± 0.018*

* *P *< 0.05 vs. control group

### PS decreased the apparent absorption of iron

**Figure 1:**
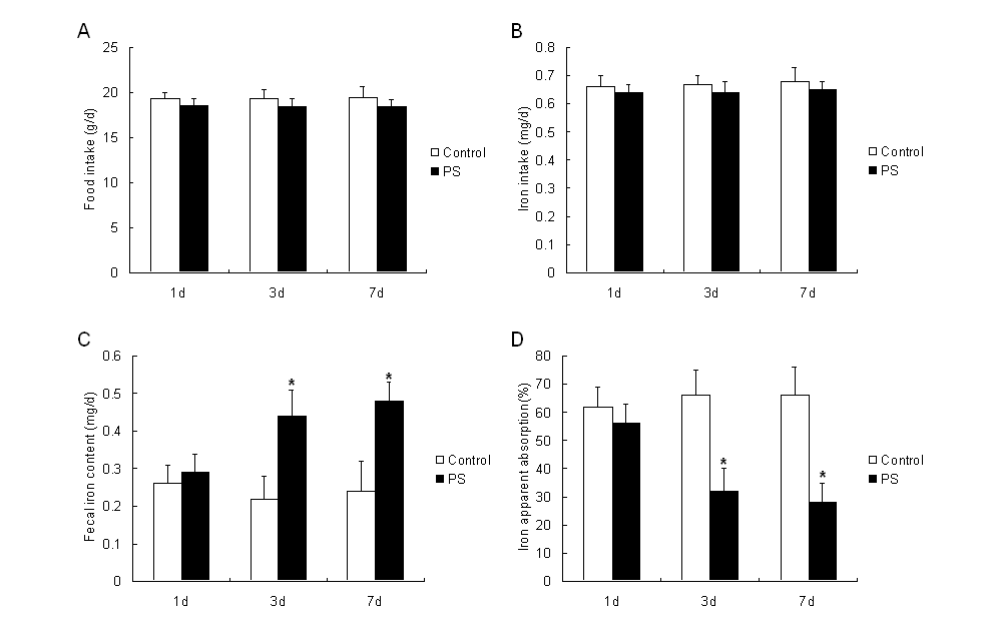
**Effect of 1 d, 3 d and 7 d PS exposures on the food intake (A), iron intake (B), fecal iron content (C) and apparent iron absorption (D)**. Values are expressed as means ± SD, n = 5. Asterisks indicates significant difference between control and PS groups, *P *< 0.05.

The apparent absorption of iron was calculated after 1 d, 3 d and 7 d PS exposure. There was no significant difference in food and iron intake between PS and control groups (Figure 1A, 1B). However, there was significant increase in fecal iron content after 3 d and 7 d PS exposure (Figure 1C). It was clear that the apparent absorption of iron in 3 d and 7 d PS groups decreased significantly (Figure 1D, *P *< 0.05).

### PS induced changes of ferritin and FPN1 protein expressions

**Figure 2:**
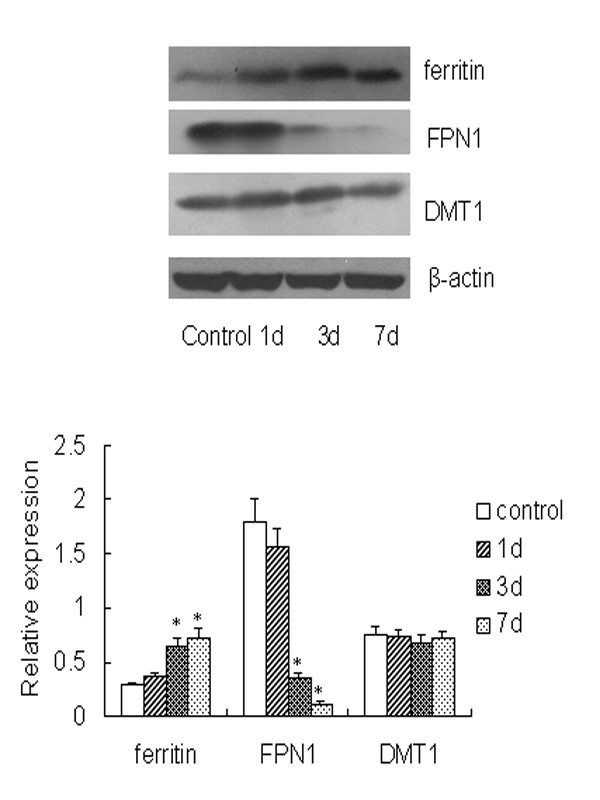
**Western blots of duodenal ferritin, FPN1 and DMT1 protein in control and PS groups on 1 d, 3 d, and 7 d**. One of five representative experiments is shown. Values are expressed as means ± SD, n = 5. Asterisks indicates significant difference between control and PS groups, *P *< 0.05.

After 1 d PS exposure, there was no significant change in DMT1, FPN1 and ferritin protein expressions. However, the FPN1 protein expression decreased, and the protein expression of ferritin increased after 3 d and 7 d PS (*P *< 0.05). The DMT1 expression did not change in two groups (Figure 2).

### Perl's staining

**Figure 3:**
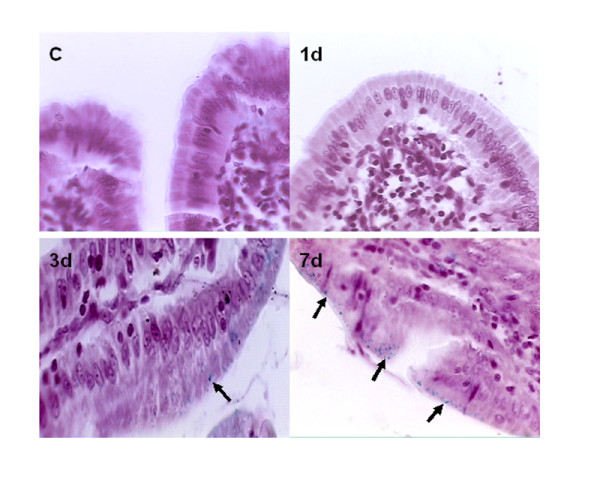
**Perl's iron staining in duodenum in control and PS groups on 1 d, 3 d, and 7 d**. Arrows show the locations of iron staining in duodenum.

Perl's iron staining revealed that no discernable staining for iron in the apical poles of villous enterocytes of control and 1 d PS groups, while there was obvious staining after 3 d and 7 d PS exposure (Figure 3).

## Discussion

Communication box, developed by Ogawa and Kuwabara, is a common model to investigate the physical and physiological changes under psychological stress [11,12]. Without direct physical stress, the box can produce an experimental anxiety based on intraspecies emotion. In our study, the hypothalamus NE, serum CORT and ACTH increased significantly after PS, which indicated that the emotional responses to foot shock activated the hypothalamic-pituitary-adrenal (HPA) axis in PS rats.

It is well known that iron absorption is regulated by a number of factors, including the level of body iron store, the rate of erythropoiesis, and hypoxia. Iron intake also could affect iron absorption in the duodenum. In the present study, there was no significant difference in the food and iron intake between control and PS groups, so the iron absorption was not related to the iron intake. A formula diet was given to exclude the factors related to feeding. However, the fecal iron contents after 3 d and 7 d PS increased significantly. Interestingly, in our previous and present study (data not shown), we found that PS didn't change the body weight in rat [7]. It was contradictory to the findings of others that found chronic stress could induce weight loss and anorexia [13,14]. We hypothesized that the difference in stress protocols might produce this discrepancy. In addition, we observed that there was no significant difference in the quantity and shape of feces between the control and PS groups. From analysis above, it was concluded that the increased fecal iron excretion led to the decreased apparent absorption of iron after PS exposure.

Overload exercise and spaceflight can lead to the decline of serum iron level. In our previous study, serum iron level was decreased after 3 d repeated psychological stress exposure before the decline of red cell count and hemoglobin (7 d) [7]. Moreover, the longer the rats suffered from PS, the greater the range that erythropoiesis was inhibited. Perl's iron staining revealed iron accumulation in the apical poles of villous enterocytes after 3 d and 7 d PS exposure. We also found that liver and spleen iron storage increased after PS in our past research [15]. It is possible that PS lead to the decreased iron absorption and iron redistribution in body induced the decreased serum iron and bone marrow iron and inhibited the synthesis of hemoglobin (Hb) and erythropoiesis.

As we all known, Iron is needed by all mammalian cells. Specialized transport mechanisms conduct iron across cellular membranes. These are regulated to ensure iron homeostasis both systemically in living organisms and within individual cells. Over the past decade, major advances have been made in identifying and characterizing the proteins involved in the transport, handling, and homeostatic regulation of iron. Molecular understanding of these processes has provided important insights into the pathophysiology of human iron disorders. Our research showed that FPN1 expression decreased after 3 d and 7 d PS, which was consistent with our previous results [9]. As demonstrated, hepcidin is synthesized in the liver as a central regulator of iron metabolism which regulates the iron absorption in intestine and the release of iron from macrophages [16,17]. And interleukin-6 (IL-6) can stimulate hepcidin expression *in vivo *and *in vivo *[18,19]. After binding to hepcidin, FPN1 is internalized and degraded, leading to the decreased export of cellular iron [17]. Our previous study showed that the liver iron storage, serum IL-6 and liver hepcidin protein expression increased after 3 d and 7 d PS, which were blocked by the injection of IL-6 antibody. Based on investigations above, we concluded that the upregulation of hepcidin and down-regulated FPN1 expression, which lead to the decreased iron absorption. As another important iron transporter, DMT1 has been extensively studied before. In present study, the iron intake had no significant difference between PS and control groups, and we also found the DMT1 expression had no change during PS, which was consistent with others' studies [20].

## Conclusion

Combined with all results mentioned above, it is possible that the FPN1 expression was downregulated through the IL-6-hepcidin axis after PS, which induced the decreased iron absorption, resulting in hypoferremia. In order to confirm it, further study would be carried out, which regard effect on iron apparent iron absorption in rats treated with anti-IL-6 antibody after ps.

## Competing interests

The authors declare that they have no competing interests.

## Authors' contributions

JC carried out the animal model and the measurement of iron apparent absorption studies. HS carried out the Perl's staining and drafted the manuscript. CC, SY, WW, and MZ carried out Western blotting and statistical analysis. ML edited and revised the manuscript and organized the study. All authors read and approved the final manuscript.

## Pre-publication history

The pre-publication history for this paper can be accessed here:

http://www.biomedcentral.com/1471-230X/9/83/prepub
